# Intelligence

**Published:** 1829-11

**Authors:** 


### INTELLIGENCE.

### MONTHLY REPORT OF DISEASES.

During the last month the metropolis has been remarkably free from any serious disease. The most frequent cases have been catarrhs and rheumatism, but they have not presented any features of sufficient importance to merit particular mention. From one case, in private practice, we are led to infer that even the external application of iodine, in the proportion of one drachm of the hydriodate of potash to one ounce of simple ointment, may be productive of serious constitutional disturbance. The ointment was applied twice a day to a suspicious tumor of the breast. During its application the patient complained of occasional loss of sight, sickness, nervous irritation, &c. These symptoms greatly subsided when the ointment was discontinued.

---

We lately mentioned that a patent had been granted to a surgeon of the name of Derbyshire for a remedy against sea-sickness. The mode of preparing it is as follows. (We give it in the untechnical dress in which it was presented to us.) Take of crude opium, two ounces; extract of henbane, two drachms; powdered mace, ten grains; hard mottled soap, two ounces: boil them in sixty ounces of soft water for half an hour, stirring them well; when cold, add one quart of spirits of wine at 60° above proof, and three drachms of spirit of ammonia. Rub a dessertspoonful of this embrocation well in and over the lower end of the breast bone, and under the left ribs, the latest time it may be convenient previous to embarkation, and again on board as soon as possible; and repeat it, if necessary.

---

*Medical Societies.*—The Medico-Chirurgical, the London Medical, and the Westminster Societies, have resumed their sittings for the present session. We shall notice any important discussion that occurs at either society.

---

We have kept open the press until the last moment, to admit the following Regulations, which are just published. We shall probably offer a few comments upon them in our next Number.

### ROYAL COLLEGE OF SURGEONS IN LONDON.

*Regulations respecting the Professional Education of Candidates for the Diploma.*

I. Candidates will be required to bring proof

1. Of being twenty-two years of age.
2. Of having been engaged six years in the acquisition of professional knowledge.
3. Of having studied Anatomy, by attendance on lectures and demonstrations, and by dissections, during two anatomical seasons. (An anatomical season is understood to extend from October to April inclusive.)
4. Of having attended two courses of lectures on Surgery, each course comprising not less than sixty lectures.
5. Of having attended lectures on the practice of Physic, on Chemistry, and on Midwifery, during six months; and on Botany and Materia Medica during three months.
6. Of having attended, during twelve months, the surgical practice of a recognised hospital in London, Dublin, Edinburgh, Glasgow, or Aberdeen; or for six months in any one of such hospitals, and twelve months in any properly constituted provincial hospital, acknowledged by the Council as competent for the purposes of instruction.

It is earnestly recommended that candidates shall have studied Anatomy, by attendance on lectures and demonstrations, and by dissections, for one anatomical season prior to their attendance on the surgical practice of an hospital.

II. Members and licentiates in Surgery of any legally constituted College of Surgeons in the united kingdom; and graduates in Surgery of any University requiring residence to obtain degrees; will be admitted for examination on producing their diploma, licence, or degree.

N.B. All certificates recognised by the Royal Colleges of Dublin and Edinburgh, or by the Universities of Glasgow and Aberdeen, as to attendance on hospitals or lectures in these places respectively, will be received.

By order,

*29th day of October*, 1829. EDMUND BELFOUR, Sec.

---

### NEW REGULATIONS OF THE SOCIETY OF APOTHECARIES.

*Regulations to be observed by Students whose attendance on Lectures commenced before January 1st*, 1829.

The Court of Examiners chosen and appointed by the Master, Wardens, and Assistants of the Society of Apothecaries, of the city of London, in pursuance of a certain Act of Parliament, "for better regulating the practice of Apothecaries throughout England and Wales," passed in the fifty-fifth year of the reign of his Majesty King George the Third, apprise all persons whom it may concern:

That every candidate for a certificate to practise as an apothecary, will be required to possess a competent knowledge of the Latin language; and, in compliance with the fourteenth and fifteenth sections of the said Act, to produce testimonials of having served an apprenticeship of not less than five years to an apothecary, of having attained the full age of twenty-one years, and of good moral conduct.

Candidates will also be required to produce testimonials of attendance on lectures and medical practice, agreeably to regulations at different times published by the Court.

Those whose attendance on lectures commenced prior to the 1st of February, 1828, will be admitted to examination after an attendance on one course of lectures on Chemistry, one course of lectures on Materia Medica, two courses of lectures on Anatomy and Physiology, two courses of lectures on the Theory and Practice of Medicine, and six months' physician's practice at an hospital, or nine months at a dispensary.

Those who began to attend lectures subsequently to the 1st of February, 1828, and previously to the 1st of October in the same year, will only be admitted to examination after the following course of study: viz. an attendance on one course of lectures on Chemistry, one course of lectures on Materia Medica and Botany, two courses of lectures on Anatomy and Physiology, two courses of lectures on the Theory and Practice of Medicine, (to be attended subsequently to the lectures on chemistry and materia medica, and to one course at least of anatomy,) and six months', at least, physician's practice at an hospital, or nine months at a dispensary; such attendance to commence subsequently to the termination of the first course of lectures on the principles and practice of medicine.

Those whose attendance on lectures commenced on or after the 1st of October, 1828, and previously to the 1st of January, 1829, will be required to produce testimonials of having attended two courses of lectures on Chemistry, two courses of lectures on Materia Medica and Botany, two courses of lectures on Anatomy and Physiology, two courses of Anatomical Demonstrations, two courses of lectures on the Theory and Practice of Medicine, (to be attended subsequently to one course of lectures on chemistry, materia medica, and anatomy,) and six months, at least, the physician's practice at an hospital, containing not less than sixty beds, or nine months at a dispensary; such attendance to commence subsequently to the termination of the first course of lectures on the principles and practice of medicine.

*Regulations to be observed by Students whose attendance on Lectures commenced since January 1st,* 1829.

The Court of Examiners chosen and appointed by the Master, Wardens, and Assistants of the Society of Apothecaries, of the city of London, in pursuance of a certain Act of Parliament, " for better regulating the practice of Apothecaries throughout England and Wales," passed in the fifty-fifth year of the reign of his Majesty King George the Third, apprise all persons whom it may concern:

That every candidate for a certificate to practise as an apothecary, will be required to possess a competent knowledge of the Latin language, and, in compliance with the fourteenth and fifteenth sections of the said Act, to produce testimonials of having served an apprenticeship of not less than five years to an apothecary, of having attained the full age of twenty-one years, and of good moral conduct; and also testimonials of having attended two courses of lectures on Chemistry, two courses of lectures on Materia Medica, Therapeutics, and Botany, two courses of lectures on Anatomy and Physiology, two courses of Anatomical Demonstrations, two courses of lectures on the Theory and Practice of Medicine, (to be attended subsequently to one course of lectures on chemistry, materia medica, and anatomy,) two courses of lectures on Midwifery and the Diseases of Women and Children, and nine months, at least, the physician's practice at an hospital, containing not less than sixty beds, or twelve months at a dispensary; such attendance to commence subsequently to the termination of the first course of lectures on the principles and practice of medicine.

Students are, moreover, earnestly recommended to attend Clinical Lectures, and diligently to avail themselves of instruction in Morbid Anatomy and Forensic Medicine.

The examination of the candidate will be as follows:

1. In translating grammatically parts of the Pharmacopœia Londinensis, and physicians' prescriptions; and, after the 1st of January, 1831, candidates will be required to translate portions of the following medical Latin authors: viz. Celsus de Medicinâ, or Gregory Conspectus Medicinæ Theoreticæ.
2. In Chemistry.
3. In Materia Medica and Therapeutics.
4. In Botany.
5. In Anatomy and Physiology.
6. In the Practice of Medicine.

N.B. Physicians' pupils, who intend to present themselves for examination, must appear personally at the beadle's office, in this Hall, and bring with them the tickets authorising their attendance on such practice, as the commencement thereof will be dated from the time of such personal appearance.

No testimonial of attendance on lectures on the principles and practice of medicine, delivered in London, or within seven miles thereof, will render a candidate eligible for examination, unless such lectures were given, and the testimonial is signed by, a fellow, candidate, or licentiate, of the Royal College of Physicians of London.

*Notice.* Every person intending to qualify himself under these regulations, to practise as an apothecary, may obtain, at the beadle's office at this Hall, (where attendance is given every day, except Sunday, from nine until two o'clock,) a printed form of certificate of all the lectures candidates are required to attend, and also of the physician's practice. The Court requests the blanks may be filled up when signed by the respective lecturers and physicians whose lectures or practice the student has attended.

Students are enjoined to observe, that, in future, these certificates, so filled up, will be required from candidates for examination, and that no other form of testimonials of attendance on lectures and medical practice will be admitted, except such certificates as have heretofore been received, if the same were obtained prior to the 1st of February, 1828; or such as bear the seal of a university or college, and the signature of the officer attached to such university or college, whose duty it is to sign certificates of attendance on the lectures given therein.

Every person offering himself for examination must give notice in writing to the clerk of the Society, on or before the Monday previously to the day of axamination; and must also, at the same time, deposit all the required testimonials at the office of the beadle.

The Court will meet in the Hall every Thursday, where candidates are required to attend at half-past four o'clock.

By order of the Court,

*London; Sept.* 1, 1829. JOHN WATSON, Sec.

For information relative to these regulations, medical students are referred to Mr. Watson, who may be seen at his residence, 43, Berners street, between the hours of nine and ten o'clock every morning, (Sunday excepted;) and for information on all other subjects connected with the "Act for better regulating the Practice of Apothecaries," application is to be made to Mr. Edmund Bacot, clerk of the Society, who attends at the Hall every Tuesday and Thursday, from one to three o'clock.

It is expressly ordered by the Court of Examiners, that no gratuity be received by any officer from any person applying for information relative to the business of this Court.

---

### MONTHLY LIST OF MEDICAL BOOKS.

### METEOROLOGICAL JOURNAL,

By Messrs. HARRIS and Co. Mathematical Instrument Makers, 50, High Holborn.

| September | Moon | Rain gauge. | Thermom. | | | Barometer. | | De Luc's Hygrom. | | Winds. | | Atmospheric Variations. | | |
|---|---|---|---|---|---|---|---|---|---|---|---|---|---|---|
| | | | 9 a.m. | max. | min. | 9 A.M. | 10P.M. | 9 A.M. | 10P.M. | 9 A.M. | 10P.M. | 9 a.m. | 2 p.m. | 10 p.m. |
| 20 | | | 53 | 58 | 46 | 29.75 | 29.68 | 62 | 62 | NE | SW | Fine | Fine | Fine |
| 21 | | .23 | 50 | 60 | 48 | .63 | .71 | 60 | 59 | W | W | Cloudy | Fine | — |
| 22 | | | 52 | 61 | 52 | .74 | .69 | 59 | 59 | WSW | SW | — | — | — |
| 23 | | | 59 | 60 | 53 | .70 | .74 | 59 | 57 | SW | SW | Foggy | Fine | — |
| 24 | | | 55 | 60 | 50 | .82 | .88 | 57 | 58 | WNW | NE | Cloudy | Fine | — |
| 25 | | | 54 | 58 | 47 | .99 | 30.04 | 59 | 59 | NNE | E | Fine | Cloudy | — |
| 26 | | | 60 | 60 | 49 | 30.06 | 30.00 | 59 | 56 | WSW | SW | Foggy | Fine | Cloudy |
| 27 | | | 56 | 62 | 47 | 29.90 | 29.68 | 58 | 58 | WSW | SSW | Show'ry | Fine | Fine |
| 28 | ● | | 54 | 56 | 45 | .75 | .79 | 60 | 56 | WNW | NNW | Fine | — | — |
| 29 | | | 49 | 58 | 43 | .84 | .96 | 54 | 54 | NW | NNW | Foggy | — | — |
| 30 | | | 52 | 59 | 47 | 30.07 | 30.11 | 56 | 56 | NNE | NNE | — | — | — |
| Oct. | | | | | | | | | | | | | | |
| 1 | | | 54 | 60 | 52 | .11 | .08 | 58 | 62 | ENE | ENE | Fine | — | — |
| 2 | | | 56 | 58 | 55 | 29.90 | 29.83 | 65 | 67 | ENE | ENE | Cloudy | Rain | Foggy |
| 3 | | .17 | 57 | 58 | 50 | .71 | .61 | 70 | 70 | SW | W | Show'ry | Rain | Fine |
| 4 | | | 54 | 58 | 47 | .68 | .54 | 67 | 67 | N | W | Fine | Fine | Cloudy |
| 5 | ☽ | | 55 | 57 | 45 | .46 | .46 | 65 | 68 | W | W | Rain | Fine | Fine |
| 6 | | .50 | 51 | 53 | 41 | .48 | .59 | 68 | 57 | WNW | NW | Fine | — | — |
| 7 | | | 42 | 41 | 34 | .40 | .36 | 58 | 57 | WNW | N | Foggy | Sn.&Ra. | Fine |
| 8 | | .63 | 40 | 43 | 35 | .68 | .91 | 57 | 56 | NNW | N | Fine | Cloudy | Fine |
| 9 | | | 46 | 49 | 44 | 30.05 | 30.18 | 56 | 57 | NNW | WNW | — | Fine | Foggy |
| 10 | | | 49 | 56 | 45 | .28 | .26 | 55 | 57 | NW | W | Foggy | Fine | Cloudy |
| 11 | | | 51 | 58 | 53 | .14 | .03 | 59 | 60 | SW | W | Fine | — | Fine |
| 12 | ○ | | 57 | 57 | 52 | .01 | .01 | 60 | 61 | NW | NW | Cloudy | Fine | Cloudy |
| 13 | | | 55 | 58 | 42 | -9.87 | 29.65 | 61 | 60 | WSW | WSW | — | Cloudy | — |
| 14 | | | 51 | 54 | 28 | .27 | .72 | 59 | 58 | NW | N | Show'ry | — | Fine |
| 15 | | .04 | 45 | 51 | 42 | 30.06 | 30.16 | 58 | 56 | N | N | Fine | — | — |
| 16 | | | 47 | 55 | 48 | 29.92 | 29.70 | 56 | 55 | SW | WSW | — | — | — |
| 17 | | | 55 | 56 | 50 | .89 | 30.00 | 54 | 59 | W | WSW | — | — | — |
| 18 | | | 56 | 60 | 53 | 30.00 | 29.97 | 61 | 62 | WSW | WSW | — | — | — |
| 19 | ☾ | | 59 | 61 | 56 | 29.94 | .90 | 64 | 65 | WSW | SW | — | Cloudy | Cloudy |

The quantity of Rain fallen in the month of September, was 2 inches and 58-100ths.

